# Relationships between Protein Intake and Renal Function in a Japanese General Population: NIPPON DATA90

**DOI:** 10.2188/jea.JE20090222

**Published:** 2010-05-05

**Authors:** Aya Higashiyama, Makoto Watanabe, Yoshihiro Kokubo, Yuu Ono, Akira Okayama, Tomonori Okamura

**Affiliations:** 1 Lifestyle-Related Disease Prevention Center, Shiga University of Medical Science, Otsu, Japan; 2 Department of Preventive Cardiology, National Cardiovascular Center, Suita, Japan; 3 The First Institute for Health Promotion and Health Care, Japan Anti-tuberculosis Association, Tokyo, Japan

**Keywords:** CKD, GFR, odds ratio, cross-sectional study, nutrition

## Abstract

**Background:**

It has been considered that reducing protein intake is one of important measures to delay the progression of chronic kidney disease (CKD). However, the relationship between protein intake and renal function is still uncertain, especially in relatively healthy general population.

**Methods:**

7404 individuals (3099 men and 4305 women) who participated in both National Survey on Circulatory Disorders and National Nutrition Survey in 1990 and were free from past history of renal diseases were included in the present study. We estimated sex-specific age- and multivariate-adjusted glomerular filtration rate (GFR) and odds ratios for the presence of CKD according to the quartiles of protein (total, animal, vegetable) intake per body weight (kg).

**Results:**

There were significant differences in each protein intake among the age groups in both men and women. Both participants with and without CKD took more protein intake than that of each recommended level. There were positive relationships between GFR and the quartiles of each protein intake in both sexes. The odds ratios for the presence of CKD were significantly decreased in the higher quartile of protein intake in women.

**Conclusions:**

The higher protein intake was associated with higher GFR in both sexes and low prevalence of CKD in women. However, further studies are needed to conclude the relationships between protein intake and renal function.

## INTRODUCTION

The hypotheses that reduction of protein intake and strict blood pressure control delays the progression of chronic kidney disease (CKD) have been tested since 1990s. Although the Modification of Diet in Renal Disease (MDRD) study did not show the statistical significance of diet intervention,^1^ a recent secondary analysis of the MDRD study, with a 6-year follow up, showed that the low protein diet with tight blood pressure control may have a beneficial effect on delaying progression in CKD Stages 3 to 4.^2^ However, other previous studies about the same study question did not show definitive results, either.^4^^–^^7^ Furthermore, in the MDRD study, the criteria for enrollment were Cre ≥ 1.4 mg/dl in men and Cre ≥ 1.2 mg/dl in women.^1^^,^^3^ Accordingly, the effect of reduced protein diet for the progression of CKD is still uncertain, especially in relatively healthy general population.

On the other hand, animal protein is considered to induce hyperfiltration,^8^^,^^9^ however, other study reported that white meat or fish provide benefits.^10^ In addition, whether vegetable protein is associated with decreased renal function is controversial.^11^^,^^12^

Accordingly, we cross-sectionally investigated the relationship between renal function and total, animal, and vegetable protein in a 7404 Japanese representative population, who participated in both the National Survey on Circulatory Disorders and the National Nutrition Survey in 1990.

## METHODS

### Study participants

The participants of the present study were 8342 community residents (3488 men and 4854 women) aged 30 and greater, who participated in both the National Survey on Circulatory Disorders, of which follow-up study is known as “*NIPPON DATA90*”,^13^^,^^14^ and the National Nutrition Survey in 1990. In these surveys, 300 areas were randomly selected form Japan, and overall population aged 30 and greater in these areas was 10 956 people. Accordingly, the participation rate was over 75%, and the participants of these surveys represent general Japanese population. Of the 8342 residents, 7404 participants (3099 men and 4305 women) who were free from past history of renal diseases were included in the present study.

### Nutritional surveys

A house-hold food weighing method was used in the National Nutrition Survey in 1990 (NNSJ90). The details of the estimation for individual nutrient intakes by calculating proportional distribution are described in another paper.^15^ Briefly, nutrient intakes of each household member were estimated by dividing household intake data of NNSJ90 proportionally using average intakes by sex and age groups calculated for NNSJ95 conducted in 1995. Protein intake was calculated as g/day, and protein intake per body weight (g/kg/day) was calculated as protein intake (g/day) divided by body weight (kg).

### Risk factor surveys

Public health nurses obtained information on smoking, alcohol drinking, past history of renal diseases and diabetes, and medication for hypertension. Blood pressure was measured by trained observers using a standard mercury sphygmomanometer on the right arm of seated participants after a sufficient period of rest. Non-fasting blood samples were obtained and the serum was separated and centrifuged soon after blood coagulation. Plasma samples were also obtained in a siliconized tube containing sodium fluoride. These samples were shipped to 1 laboratory (SRL, Tokyo, Japan) for blood measurements. Serum creatinine (mg/dl) was measured using the alkaline picric acid method (Jaffe). GFR (ml/min per 1.73 m^2^) was calculated using the abbreviated equation developed at the Cleveland Clinic laboratory for the Modification of Diet in Renal Disease (MDRD) study: 186 × [serum creatinine (mg/dl)] − 1.154 × [age (years)] − 0.203 × [0.742 if female].^16^ Imai et al recommend to multiply the value calculated using the abbreviated MDRD equation by 0.881 for Japanese.^17^ Accordingly, we calculated GFR by multiplying 0.881. Referring to the NKF classification of CKD,^18^^,^^19^ the participants were classified into the 2 groups: GFR ≥ 60 and GFR < 60. The latter group was defined as CKD.

### Statistical analysis

The statistical analyses were based on the data for sex-specific quartiles of total, animal, and vegetable protein intake per body weight (kg). To compare the characteristics, we used analysis of variance or t test for continuous variables and chi-square test for categorical variables. Age- and multivariate-adjustment was performed by analysis of covariance or logistic regression model.

We estimated age- and multivariate-adjusted GFR and odds ratios of quartiles of protein intake for the presence of CKD, compared to the lowest quartile of the protein intake. The confounding variables included age (linear), systolic blood pressure, serum total cholesterol, HbA1c, current smoking, and current alcohol drinking. The same analyses were performed after excluding the participants with past history of hypertension and diabetes.

The statistical package SPSS 15.0J for Windows (SPSS, Tokyo, Japan) performed these analyses. All probability values were 2-tailed and the significance level was established at *P* < 0.05.

## RESULTS

In men, the mean GFR was 83.7 ml/min per 1.73 m^2^, and the mean protein intake was 1.49 g/kg/day in total, 0.76 g/kg/day in animal, and 0.73 g/kg/day in vegetable protein. In women, the mean GFR was 84.4 ml/min per 1.73 m^2^, the mean protein intake was 1.44 g/kg/day in total, 0.73 g/kg/day in animal, and 0.71 g/kg/day in vegetable protein.

Table 1 shows the total, animal, and vegetable protein intake according to the age groups by sex. There were significant differences in protein intake among the age groups both in men and women. In men, the protein intake was the highest in 50–59 age group, and the lowest in 80– age group except for protein intake per body weight (g/day/kg). In women, the protein intake was the highest in 40–49 or 50–59 age groups except for vegetable protein intake, and the lowest in 80– age group except for protein intake per body weight (g/day/kg). In addition, we present the sex-specific mean total protein intake (g/kg/day) according to the presence of CKD. In men, the mean (± standard deviation) total protein intake was 1.49 ± 0.36 among the individuals without CKD, and 1.46 ± 0.36 among those with CKD (*P* = 0.35). In women, it was 1.45 ± 0.35 among the individuals without CKD, and 1.35 ± 0.36 among those with CKD (*P* < 0.001) (data not shown in the table).

**Table 1.** Total-, animal-, vegetable protein intake according to age group: NIPPON DATA90

	Age						*P* value
	
	30–39	40–49	50–59	60–69	70–79	80–	
**Men (*n* = 3099)**							
*n*	582	741	712	633	350	81	
Total protein intake (g/day)	88.8 ± 18.3	93.2 ± 18.2	97.1 ± 20.7	87.4 ± 18.3	79.0 ± 18.1	73.9 ± 16.1	<0.001
Total protein intake per body weight (g/day/kg)	1.39 ± 0.32	1.46 ± 0.34	1.60 ± 0.38	1.52 ± 0.37	1.44 ± 0.38	1.44 ± 0.33	<0.001
Animal protein intake (g/day)	46.0 ± 13.5	49.2 ± 13.9	50.5 ± 15.6	43.6 ± 13.5	38.8 ± 13.1	36.3 ± 12.5	<0.001
Animal protein intake per body weight (g/day/kg)	0.72 ± 0.22	0.77 ± 0.24	0.83 ± 0.26	0.76 ± 0.25	0.70 ± 0.25	0.71 ± 0.25	<0.001
Vegetable protein intake (g/day)	42.8 ± 8.7	43.9 ± 8.5	46.5 ± 9.5	43.9 ± 9.1	40.4 ± 9.0	37.7 ± 7.8	<0.001
Vegetable protein intake per body weight (g/day/kg)	0.67 ± 0.16	0.69 ± 0.16	0.77 ± 0.19	0.77 ± 0.19	0.74 ± 0.19	0.74 ± 0.17	<0.001

**Women (*n* = 4305)**							
*n*	938	1051	925	807	453	131	
Total protein intake (g/day)	71.9 ± 12.9	78.1 ± 14.9	78.1 ± 16.9	72.9 ± 16.4	64.6 ± 14.3	63.0 ± 13.6	<0.001
Total protein intake per body weight (g/day/kg)	1.39 ± 0.31	1.48 ± 0.34	1.49 ± 0.38	1.44 ± 0.37	1.37 ± 0.35	1.44 ± 0.41	<0.001
Animal protein intake (g/day)	37.1 ± 9.9	41.1 ± 11.2	39.7 ± 12.3	35.8 ± 11.6	31.1 ± 10.5	30.0 ± 9.5	<0.001
Animal protein intake per body weight (g/day/kg)	0.72 ± 0.21	0.78 ± 0.24	0.76 ± 0.25	0.71 ± 0.24	0.66 ± 0.23	0.69 ± 0.25	<0.001
Vegetable protein intake (g/day)	35.0 ± 6.4	37.0 ± 7.1	38.5 ± 8.1	37.2 ± 8.3	33.7 ± 7.2	33.1 ± 7.1	<0.001
Vegetable protein intake per body weight (g/day/kg)	0.67 ± 0.15	0.70 ± 0.16	0.73 ± 0.19	0.74 ± 0.20	0.71 ± 0.18	0.76 ± 0.21	<0.001

Data are presented as mean ± standard deviation.

Table 2 shows the age- and multivariate-adjusted GFR and the odds ratio for CKD according to the quartiles of total protein intake (g/kg/day). There were positive significant relationships between the total protein intake and the multivariate-adjusted GFR in both sexes. And there were significant negative relationships between the quartiles of total protein intake and the odds ratios for CKD in women.

**Table 2.** Age- and multivariate-adjusted GFR and Odds ratio for CKD according to the quartiles of total protein intake (g/day/kg): NIPPON DATA90

	Total protein intake (g/day/kg)				*P* value
	
	1 (low)	2	3	4 (high)	
**Men (*n* = 3099)**					
*n*	770	791	756	782	
Age (years)	52 ± 15	52 ± 14	53 ± 13	56 ± 12	<0.001
Mean total protein intake (g/day/kg)	1.07 ± 0.13	1.35 ± 0.06	1.57 ± 0.07	1.97 ± 0.26	<0.001
Body mass index (kg/m^2^)	24.7 ± 3.1	23.3 ± 2.7	22.4 ± 2.7	21.3 ± 2.5	<0.001
Systolic blood pressure (mm Hg)	139 ± 20	137 ± 20	137 ± 21	137 ± 20	0.09
Diastolic blood pressure (mm Hg)	85 ± 12	84 ± 12	84 ± 12	82 ± 12	<0.001
Total cholesterol (mg/dl)	203 ± 38	199 ± 37	196 ± 36	196 ± 35	<0.01
HbA1c (%)	5.0 ± 0.7	5.0 ± 0.8	5.0 ± 0.7	5.0 ± 0.8	0.89
Alcohol drinking (%)	55.6	56.8	60.6	59.3	0.17
Current smoker (%)	55.6	54.4	56.0	55.9	0.91

GFR (age-adjusted)^a^	81.8 (80.7–82.8)	82.1 (81.0–83.1)	83.4 (82.3–84.5)	87.6 (86.5–88.7)	<0.001
GFR (multivariate-adjusted)^a^	82.1 (81.0–83.1)	82.1 (81.1–83.2)	83.2 (82.1–84.3)	87.4 (86.4–88.5)	<0.001
Odds ratio for CKD (age-adjusted)^b^	Reference	1.02 (0.64–1.64)	1.25 (0.79–1.98)	0.65 (0.39–1.08)	0.14
Odds ratio for CKD (multivariate-adjusted)^b^	Reference	1.00 (0.62–1.62)	1.31 (0.82–2.08)	0.70 (0.42–1.16)	0.25

**Women (*n* = 4305)**					
*n*	1070	1096	1072	1067	
Age (years)	53 ± 15	52 ± 14	52 ± 14	53 ± 13	<0.01
Mean total protein intake (g/day/kg)	1.03 ± 0.13	1.3 ± 0.06	1.52 ± 0.68	1.92 ± 0.25	<0.001
Body mass index (kg/m^2^)	25.1 ± 3.5	23.2 ± 2.9	22.1 ± 2.8	20.9 ± 2.6	<0.001
Systolic blood pressure (mm Hg)	137 ± 22	134 ± 21	132 ± 20	131 ± 20	<0.001
Diastolic blood pressure (mm Hg)	82 ± 12	80 ± 12	79 ± 11	78 ± 11	<0.001
Total cholesterol (mg/dl)	209 ± 41	207 ± 40	205 ± 38	206 ± 36	<0.05
HbA1c (%)	5.0 ± 0.8	4.9 ± 0.7	4.9 ± 0.7	4.8 ± 0.6	<0.001
Alcohol drinking (%)	6.5	6.8	6.3	6.7	0.95
Current smoker (%)	11.0	7.8	9.3	9.2	0.08

GFR (age-adjusted)^a^	83.0 (82.0–84.0)	83.3 (82.4–84.3)	85.5 (84.5–86.4)	85.7 (84.7–86.6)	<0.001
GFR (multivariate-adjusted)^a^	83.2 (82.2–84.2)	83.4 (82.4–84.3)	85.4 (84.4–86.3)	85.5 (84.6–86.5)	<0.001
Odds ratio for CKD (age-adjusted)^b^	Reference	0.83 (0.61–1.13)	0.55 (0.39–0.78)	0.51 (0.36–0.72)	<0.001
Odds ratio for CKD (multivariate-adjusted)^b^	Reference	0.85 (0.62–1.16)	0.57 (0.41–0.81)	0.54 (0.38–0.77)	<0.001

Data are presented as mean ± standard deviation. Values in parentheses indicate 95% confidence interval.

Analysis of variance. ^a^Analysis of covariance. ^b^Logistic regression analysis.

Multivariate-adjustment: age, systolic blood pressure, serum total cholesterol, HbA1c, smoking (current or non-current), alcohol drinking (current or non-current) were adjusted.

Table 3 shows the age- and multivariate-adjusted GFR and the odds ratio for CKD according to the quartiles of animal protein intake (g/kg/day). There were positive significant relationships between the quartiles of the animal protein intake and the multivariate-adjusted GFR in both sexes. In women, there was a significant decrease of the odds ratio for CKD in the highest animal protein intake group.

**Table 3.** Age- and multivariate-adjusted GFR and Odds ratio for CKD according to the quartiles of animal protein intake (g/day/kg): NIPPON DATA90

	Animal protein intake (g/day/kg)				*P* value
	
	1 (low)	2	3	4 (high)	
**Men (*n* = 3099)**					
*n*	786	777	780	756	
Age (years)	54 ± 15	52 ± 14	53 ± 13	54 ± 12	<0.001
Mean total protein intake (g/day/kg)	0.49 ± 0.08	0.66 ± 0.04	0.81 ± 0.05	1.11 ± 0.18	<0.001
Body mass index (kg/m^2^)	24.2 ± 3.3	23.2 ± 2.8	22.5 ± 2.7	21.8 ± 2.8	<0.001
Systolic blood pressure (mm Hg)	140 ± 20	138 ± 21	136 ± 20	137 ± 19	<0.01
Diastolic blood pressure (mm Hg)	85 ± 12	84 ± 12	83 ± 12	83 ± 11	<0.01
Total cholesterol (mg/dl)	198 ± 39	199 ± 37	196 ± 34	200 ± 37	0.33
HbA1c (%)	5.0 ± 0.7	5.0 ± 0.8	5.0 ± 0.7	5.0 ± 0.9	0.49
Alcohol drinking (%)	53.2	57.5	60.1	61.5	<0.01
Current smoker (%)	54.6	57.0	55.9	54.2	0.68

GFR (age-adjusted)^a^	82.7 (81.6–83.8)	83.0 (81.9–84.1)	83.6 (82.5–84.7)	85.6 (84.5–86.7)	<0.01
GFR (multivariate-adjusted)^a^	82.8 (81.7–83.9)	83.0 (82.0–84.1)	83.5 (82.4–84.5)	85.6 (84.5–86.7)	<0.01
Odds ratio for CKD (age-adjusted)^b^	Reference	0.81 (0.50–1.31)	0.96 (0.60–1.52)	0.90 (0.57–1.44)	0.80
Odds ratio for CKD (multivariate-adjusted)^b^	Reference	0.81 (0.50–1.31)	0.97 (0.61–1.54)	0.93 (0.58–1.48)	0.89

**Women (*n* = 4305)**					
*n*	1055	1076	1091	1083	
Age (years)	55 ± 15	52 ± 14	52 ± 14	51 ± 13	<0.001
Mean total protein intake (g/day/kg)	0.46 ± 0.08	0.63 ± 0.04	0.78 ± 0.05	1.05 ± 0.17	<0.001
Body mass index (kg/m^2^)	24.5 ± 3.5	23.3 ± 3.1	22.3 ± 2.9	21.3 ± 2.8	<0.001
Systolic blood pressure (mm Hg)	138 ± 21	134 ± 21	133 ± 20	130 ± 20	<0.001
Diastolic blood pressure (mm Hg)	82 ± 12	80 ± 12	79 ± 11	78 ± 11	<0.001
Total cholesterol (mg/dl)	208 ± 41	206 ± 40	206 ± 37	206 ± 37	0.74
HbA1c (%)	5.0 ± 0.8	4.9 ± 0.7	4.9 ± 0.8	4.8 ± 0.6	<0.001
Alcohol drinking (%)	4.8	7.2	7.3	7.0	0.07
Current smoker (%)	8.9	9.0	9.3	10.1	0.79

GFR (age-adjusted)^a^	83.5 (82.5–84.5)	83.8 (82.8–84.7)	84.2 (83.2–85.2)	86.0 (85.0–86.9)	<0.01
GFR (multivariate-adjusted)^a^	83.5 (82.6–84.5)	83.8 (82.8–84.8)	84.2 (83.2–85.2)	85.9 (84.9–86.8)	<0.01
Odds ratio for CKD (age-adjusted)^b^	Reference	0.78 (0.57–1.07)	0.78 (0.57–1.07)	0.51 (0.35–0.73)	<0.001
Odds ratio for CKD (multivariate-adjusted)^b^	Reference	0.78 (0.57–1.08)	0.78 (0.57–1.57)	0.53 (0.37–0.76)	<0.01

Data are presented as mean ± standard deviation. Values in parentheses indicate 95% confidence interval.

Analysis of variance. ^a^Analysis of covariance. ^b^Logistic regression analysis.

Multivariate-adjustment: age, systolic blood pressure, serum total cholesterol, HbA1c, smoking (current or non-current), alcohol drinking (current or non-current) were adjusted.

Table 4 shows the age- and multivariate-adjusted GFR and the odds ratio for CKD according to the quartiles of vegetable protein intake (g/kg/day). In both men and women, the significant positive relationship between the vegetable protein intake and the multivariate-adjusted GFR was observed. In women, there was a significant decrease of the odds ratio for CKD in the highest vegetable protein intake group.

**Table 4.** Age- and multivariate-adjusted GFR and Odds ratio for CKD according to the quartiles of vegetable protein intake (g/day/kg): NIPPON DATA90

	Plant protein intake (g/day/kg)				*P* value
	
	1(low)	2	3	4(high)	
**Men (*n* = 3099)**					
*n*	769	799	770	761	
Age (years)	50 ± 14	52 ± 13	54 ± 13	57 ± 13	<0.001
Mean total protein intake (g/day/kg)	0.52 ± 0.07	0.66 ± 0.03	0.77 ± 0.03	0.97 ± 0.14	<0.001
Body mass index (kg/m^2^)	24.7 ± 3	23.5 ± 2.8	22.3 ± 2.6	21.2 ± 2.4	<0.001
Systolic blood pressure (mm Hg)	138 ± 19	138 ± 21	137 ± 20	138 ± 21	0.86
Diastolic blood pressure (mm Hg)	85 ± 12	84 ± 12	83 ± 11	82 ± 11	<0.001
Total cholesterol (mg/dl)	205 ± 38	199 ± 36	197 ± 37	192 ± 34	<0.001
HbA1c (%)	5.0 ± 0.7	5.1 ± 0.8	5.0 ± 0.8	5.0 ± 0.7	0.39
Alcohol drinking (%)	57.0	62.0	57.5	55.6	0.06
Current smoker (%)	55.4	55.9	53.6	56.8	0.65

GFR (age-adjusted)^a^	80.1 (79.0–81.2)	83.0 (82.0–84.19	83.8 (82.8–84.9)	88.0 (86.9–89.1)	<0.001
GFR (multivariate-adjusted)^a^	80.5 (79.4–81.6)	83.0 (82.0–84.1)	83.8 (82.8–84.9)	87.6 (86.5–88.7)	<0.01
Odds ratio for CKD (age-adjusted)^b^	Reference	0.89 (0.55–1.44)	0.86 (0.53–1.38)	0.63 (0.39–1.02)	0.06
Odds ratio for CKD (multivariate-adjusted)^b^	Reference	0.89 (0.55–1.44)	0.90 (0.56–1.45)	0.70 (0.42–1.14)	0.16

**Women (*n* = 4305)**					
*n*	1047	1073	1096	1089	
Age (years)	51 ± 14	51 ± 14	52 ± 14	55 ± 14	<0.001
Mean total protein intake (g/day/kg)	0.51 ± 0.06	0.64 ± 0.03	0.75 ± 0.03	0.95 ± 0.13	<0.001
Body mass index (kg/m^2^)	25 ± 3.5	23.2 ± 2.9	22.2 ± 2.7	21 ± 2.6	<0.001
Systolic blood pressure (mm Hg)	135 ± 22	134 ± 21	133 ± 20	133 ± 21	0.08
Diastolic blood pressure (mm Hg)	81 ± 12	80 ± 12	79 ± 11	78 ± 11	<0.001
Total cholesterol (mg/dl)	210 ± 40	208 ± 41	205 ± 38	205 ± 37	<0.01
HbA1c (%)	5.0 ± 0.8	4.9 ± 0.8	4.9 ± 0.7	4.9 ± 0.6	<0.01
Alcohol drinking (%)	7.2	7.8	6.5	5.0	<0.05
Current smoker (%)	13.0	8.8	7.8	7.9	<0.001

GFR (age-adjusted)^a^	82.8 (81.8–83.8)	83.7 (82.7–84.6)	85.1 (84.2–86.1)	85.7 (84.8–86.7)	<0.001
GFR (multivariate-adjusted)^a^	83.0 (82.1–84.1)	83.8 (82.8–84.7)	85.0 (84.1–86.0)	85.5 (84.5–86.5)	<0.01
Odds ratio for CKD (age-adjusted)^b^	Reference	0.84 (0.60–1.18)	0.84 (0.60–1.17)	0.55 (0.39–0.77)	<0.01
Odds ratio for CKD (multivariate-adjusted)^b^	Reference	0.86 (0.61–1.20)	0.87 (0.62–1.22)	0.59 (0.41–0.83)	<0.01

Data are presented as mean ± standard deviation. Values in parentheses indicate 95% confidence interval.

Analysis of variance. ^a^Analysis of covariance. ^b^Logistic regression analysis.

Multivariate-adjustment: age, systolic blood pressure, serum total cholesterol, HbA1c, smoking (current or non-current), alcohol drinking (current or non-current) were adjusted.

When we adjusted for age by 10-year increment or performed the same analyses in each groups divided by more than and under 65 years old, the above-mentioned results were not substantially affected.

## DISCUSSION

In the present study, there were positive relationships between GFR and the quartiles of the total, animal, and vegetable protein intake in both men and women, and most of these relationships were statistically significant. And the odds ratio for the presence of CKD was significantly decreased in the higher quartile groups of each protein intake in women.

For the general characteristics of the nutrition data in the present study, the mean total protein intake was different among the age groups. In men, total protein intake per day was gradually reduced by approximately 11–23% by 70 year of age. This tendency was almost same with the results of the previous study performed in the United States population, which reported that there is a gradual reduction in absolute protein intake by approximately 15% by 70 yr age.^20^ Additionally, some previous studies reported that protein intake is 30 to 50% lower in women than that in men.^21^^,^^22^ In the present study, 17 to 36% reduction in the total protein intake of women compared to that of men in each age groups, although the percentage of the reduction in the present study was smaller than that in the United States. This sex difference of protein intake between the two countries is not explained by the sex difference of BMI, because the ratio of BMI (women/men) was almost the same in both countries according to the INTERMAP study (BMI ratio; the United States 98.6%, Japan 97.9%).^23^ When we compared protein intake per body weight (g/kg/day) by sex, the sex difference was 5.0–6.9% in the present study. The variation in protein intake parallels that in body composition, especially muscle mass, and factoring dietary protein intake by body weight does not make statistical adjustment for the variation related to age and gender.^24^ The cause of variation in protein intakes among age and sex was not determined in the present study, however, it is important to take into account of age, sex and race when we consider the protein intake.

In the present study, among the participants without CKD, the mean total protein intake was 1.49 g/kg/day in men and 1.45 g/kg/day in women. Even among the individuals with CKD, the mean total protein intake among those in the present study was 1.46 g/kg/day in men and 1.35 g/kg/day in women. According to the clinical guideline,^25^ the recommended protein intake is less than 0.8 g/kg/day among the individuals with normal kidney function (GFR ≥ 90), and 0.60–0.75 g/kg/day among those with decreased GFR without dialysis.^25^^–^^27^ The Japanese Society of Nephrology recommended 0.60–0.80 g/kg/day protein intake for the individuals with CKD stage 3–5.^28^ Accordingly, the community residents in Japan had more protein intake than that of the recommended level. The future study, such as cohort study, is needed to examine the recommended protein intake for Japanese community residents with normal kidney function to prevent CKD. In addition, we should continuously give information about the presence of the recommended protein intake for CKD patients.

Brenner et al postulated that protein intake increases renal plasma blood flow and GFR leading to glomerular hyperfiltration and hypertension, and over time, results in kidney injury.^29^ It is well known that a high protein load acutely increases GFR, renal plasma flow, and proteinuria,^30^ and the Kidney Dialysis Outcome Quality Initiative (KDOQI) Clinical practice guideline for Chronic Kidney Disease: Evaluation, Classification, and Stratification by the National Kidney Foundation suggested low protein intake.^18^ In the MDRD Study, the effect of dietary protein restriction on the progression of CKD remains uncertain despite decades of study. Levey et al considered that one of the reasons for the uncertainty is that the MDRD Study may not have been of sufficient duration to observe a beneficial effect.^2^ Indeed, with a 6-year follow up, they showed that the low protein diet with tight blood pressure control suggested a beneficial effect on delaying progression in CKD Stages 3 to 4.^2^ As we mentioned, the high GFR in high total protein intake group might reflect hyperfiltration in kidneys in the present study. We need long-term follow-up studies to conclude this issue, because the progression of CKD may need long duration as Levey et al indicated.^2^ However, we cannot conclude the higher GFR reflects hyperfiltration or just the better kidney function in the high protein intake group.

In the present study, the relationships between GFR and total, animal, and vegetable-protein intake were separately assessed. Kitazato et al reported that vegetable protein may be excluded from lists of restriction in low protein diet therapy in trials in which they added vegetable protein to animal protein and observed renal hemodynamics.^11^ On the other hand, Orita et al reported that vegetable protein with the same amino-acid composition could enhance the GFR in healthy individuals as much as animal protein.^12^ In the present study, both in men and women, there was a similar positive relationship between GFR and both animal and vegetable protein intake. From the result of the present study, both animal and vegetable protein might be associated with higher GFR.

The present study has several limitations. First, the present study is a cross-sectional study. Accordingly, the association between each protein intake and renal function does not prove causal relation. Second, the nutritional data is calculated by a combination method of household-based food weighing record and an approximation of proportions by which family members shared each dish or food in the household. Accordingly, the nutritional data might not accurately reflect the individual nutritional intake. However, we believe this method is the best available because the tendency of protein intake according to the age groups by sex was almost similar to the results in the recent National Nutrition Survey based on the individual data.^31^

In conclusion, there were positive relationships between GFR and total, animal, and vegetable protein. And the risk of CKD tended to be lower in higher protein intake groups. However, further cohort study with detailed nutrition survey is needed to conclude about the association between protein intake and renal function.
